# A porcine study of ultrasound-guided versus fluoroscopy-guided placement of endovascular balloons in the inferior vena cava (REBOVC) and the aorta (REBOA)

**DOI:** 10.1136/tsaco-2022-001075

**Published:** 2023-05-12

**Authors:** Maria B Wikström, Jens Åström, Anna Stene Hurtsén, Tal M Hörer, Kristofer F Nilsson

**Affiliations:** 1 Örebro University School of Medical Sciences, Faculty of Medicine and Health, Örebro, Sweden; 2 Centrum för Klinisk Forskning, Region Värmland, Karlstad, Sweden; 3 Emergency Department, Arvika Hospital, Arvika, Sweden; 4 Department of Anesthesiology, Falun Hospital, Region Dalarna, Sweden; 5 Department of Cardiothoracic and Vascular Surgery, Örebro University Hospital, Örebro, Sweden

**Keywords:** shock, hemorrhagic, multiple trauma, veins, ultrasonography

## Abstract

**Objectives:**

In fluoroscopy-free settings, alternative safe and quick methods for placing resuscitative endovascular balloon occlusion of the aorta (REBOA) and resuscitative endovascular balloon occlusion of the inferior vena cava (REBOVC) are needed. Ultrasound is being increasingly used to guide the placement of REBOA in the absence of fluoroscopy. Our hypothesis was that ultrasound could be used to adequately visualize the suprahepatic vena cava and guide REBOVC positioning, without significant time-delay, when compared with fluoroscopic guidance, and compared with the corresponding REBOA placement.

**Methods:**

Nine anesthetized pigs were used to compare ultrasound-guided placement of supraceliac REBOA and suprahepatic REBOVC with corresponding fluoroscopic guidance, in terms of correct placement and speed. Accuracy was controlled by fluoroscopy. Four intervention groups: (1) fluoroscopy REBOA, (2) fluoroscopy REBOVC, (3) ultrasound REBOA and (4) ultrasound REBOVC. The aim was to carry out the four interventions in all animals. Randomization was performed to either fluoroscopic or ultrasound guidance being used first. The time required to position the balloons in the supraceliac aorta or in the suprahepatic inferior vena cava was recorded and compared between the four intervention groups.

**Results:**

Ultrasound-guided REBOA and REBOVC placement was completed in eight animals, respectively. All eight had correctly positioned REBOA and REBOVC on fluoroscopic verification. Fluoroscopy-guided REBOA placement was slightly faster (median 14 s, IQR 13–17 s) than ultrasound-guided REBOA (median 22 s, IQR 21–25 s, p=0.024). The corresponding comparisons of the REBOVC groups were not statistically significant, with fluoroscopy-guided REBOVC taking 19 s, median (IQR 11–22 s) and ultrasound-guided REBOVC taking 28 s, median (IQR 20–34 s, p=0.19).

**Conclusion:**

Ultrasound adequately and quickly guide the placement of supraceliac REBOA and suprahepatic REBOVC in a porcine laboratory model, however, safety issues must be considered before use in trauma patients.

**Level of evidence:**

Prospective, experimental, animal study. Basic science study.

**WHAT IS ALREADY KNOWN ON THIS TOPIC**

- Resuscitative endovascular balloon occlusion of the vena cava inferior (REBOVC) is a new tool to provide hemorrhage control from vena cava injuries, until definitive surgical treatment can be achieved; however, it is unknown whether REBOVC can be correctly positioned by using ultrasound.

**WHAT THIS STUDY ADDS**

- This porcine study found that ultrasound can be used to guide correct and fast positioning of REBOVC in the suprahepatic inferior vena cava, comparable to fluoroscopic guidance.

**HOW THIS STUDY MIGHT AFFECT RESEARCH PRACTICE OR POLICY**

- These findings are expected to be translated to humans, but further research, for example, on safety issues, is recommended in human models before widespread clinical use.

## Background

Endovascular balloon occlusion is increasingly used in the management of life-threatening bleeding in both vascular surgery and trauma, and to an increasing extent in other causes of hemorrhagic shock.^1^ Resuscitative endovascular balloon occlusion of the aorta (REBOA) has been shown to be a valid alternative to emergency thoracotomy and open aortic cross-clamping in patients in extremis due to severe hemorrhagic shock.^2^ Endovascular balloons can stabilize patients in hemorrhagic shock and increase the probability of reaching an operating suite, where definitive surgical management of the injury can be achieved.^3–5^

Retrohepatic venous injuries are rare, but carry high mortality rates (30%–80%).^6–10^ In the surgical management of life-threatening retrohepatic venous bleeding, total hepatic vascular isolation has traditionally been used, that is, ‘the Heaney Maneuver’ with cross-clamping of the aorta, the infrahepatic and suprahepatic vena cava and ‘the portal triad’ in ligamentum hepatoduodenale.^11^ However, recent case reports have indicated that endovascular balloon occlusion of the vena cava (REBOVC) may present an alternative to open inferior caval venous and aortic cross-clamping.^3 4 12^ Previous porcine studies have suggested that a combination of REBOVC and REBOA may provide hemodynamic stability in hemorrhagic shock.^13 14^

Blind REBOA placement, using external anatomical landmarks or standard fixed distances, is commonly used, but has its limitations.^15–19^ Fluoroscopy may guide the correct positioning of the endovascular balloons, but may not be an available option in emergency situations, austere military and prehospital settings, compared with the prehospital often available point-of-care ultrasound.^20–22^ The use of ultrasound to guide REBOA placement is not new, but the use of ultrasound for guidance of REBOVC has not previously been reported.^21–23^ The primary aim of this study was to examine if ultrasound could be used to guide the positioning of the REBOVC at the correct level. The secondary aim was to compare ultrasound-guided placement of REBOVC and REBOA placement with fluoroscopy guidance, in terms of accuracy and speed. We opted to compare the two different imaging methods with each other, rather than comparing an imaging method with a blind method.

## Methods

### Animals

A prospective experimental animal study was performed in a research laboratory at a University Hospital. Ten pigs (a cross-breed between Hampshire, England Yorkshire and Swedish country breed, 3 months old, mean weight 30 kg, range 26.5–36.5 kg, gender ratio of approximately 1:1) were used. The pigs were allowed to eat and drink freely at the local farm before transportation to the laboratory.

The project was led by a skilled scientist schooled in animal experimentation and was supervised by a veterinarian. The ARRIVE guidelines for reporting animal studies and a legislative order on the protection of animals used for scientific purposes were followed.^24 25^

### Anesthesia

The administration of anesthetic drugs and the management of respiration and euthanasia have been previously reported.^13^ However, in the present study remifentanil at 0.5 µg/kg/min was used instead of fentanyl for analgesia. In brief, the animals were given azaperone intramuscularly prior to transfer from the farm to the laboratory. On arrival at the laboratory, a combination of tiletamine, zolazepam and azaperone was administered intramuscularly to induce general anesthesia. Continuous intravenous infusions of propofol and remifentanil were then given to preserve general anesthesia. Atropine intramuscularly was administered before endotracheal intubation. Following intubation, the respiratory frequency was modified for normoventilation and a constant ventilation at tidal volume 10 mL/kg was predetermined using a ventilator in volume-controlled mode. Uninterrupted intravenous infusions of Ringer’s acetate (10 mL/kg/hour) and 5% glucose solutions (1 mL/kg/hour) were delivered throughout the experiments to compensate for basal fluid losses. Body temperature was kept at 37.5°C–39.5°C using thermal blankets. When the experiments were finished, and while still maintaining general anesthesia, potassium chloride was given intravenously to rapidly induce cardiac arrest and euthanize the animals. Circulatory arrest was confirmed by blood pressure and end-tidal carbon dioxide recordings.

### Surgical preparation

A comprehensive description of the surgical arrangements has been reported recently.^13^ In brief, a pulmonary arterial catheter was introduced into the right external jugular vein for the monitoring of pulmonary arterial pressure, body temperature and cardiac output. In the left external jugular vein, a sheath was introduced for the infusion of solutions and drugs. A sheath was placed in the right common carotid artery by open surgical exposure to enable hemodynamic monitoring and arterial blood sampling. The right femoral artery was exposed for placement of the REBOA, and the right femoral vein was exposed and cannulated for placement of the REBOVC. The urinary bladder was continuously emptied through a catheter.

### Study protocol

**Figure 1:**
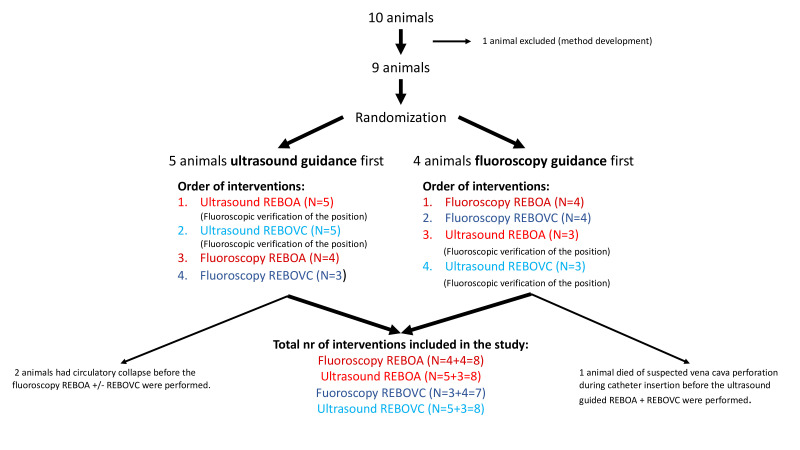
Study protocol and animal inclusion where nine anesthetized pigs were randomized in a cross-over fashion to ultrasound-guided and fluoroscopy-guided balloon placement of resuscitative endovascular balloon occlusion of the aorta (REBOA) and supraheptic vena cava (REBOVC).

After surgical preparation, 5000 E Heparin was administered intravenously and the animals were allowed 1 hour of rest. Randomization was made by blindly drawing lots from a ballot, to either endovascular balloon placement guided by fluoroscopy (Philips BV 300, Stockholm, Sweden) first, followed by ultrasound guidance (Pure Wave, Philips and V-Scan Air, GE Healthcare), or endovascular balloon placement guided by ultrasound first, followed by fluoroscopy guidance. The animals were randomized to placement of the REBOA and REBOVC guided by either ultrasound first or fluoroscopy first (figure 1). The aim was to, in partly randomized order, perform the placements using all four imaging options in every animal: (a) REBOA by fluoroscopy, (b) REBOVC by fluoroscopy, (c) REBOA by ultrasound and (d) REBOVC by ultrasound.

**Figure 2:**
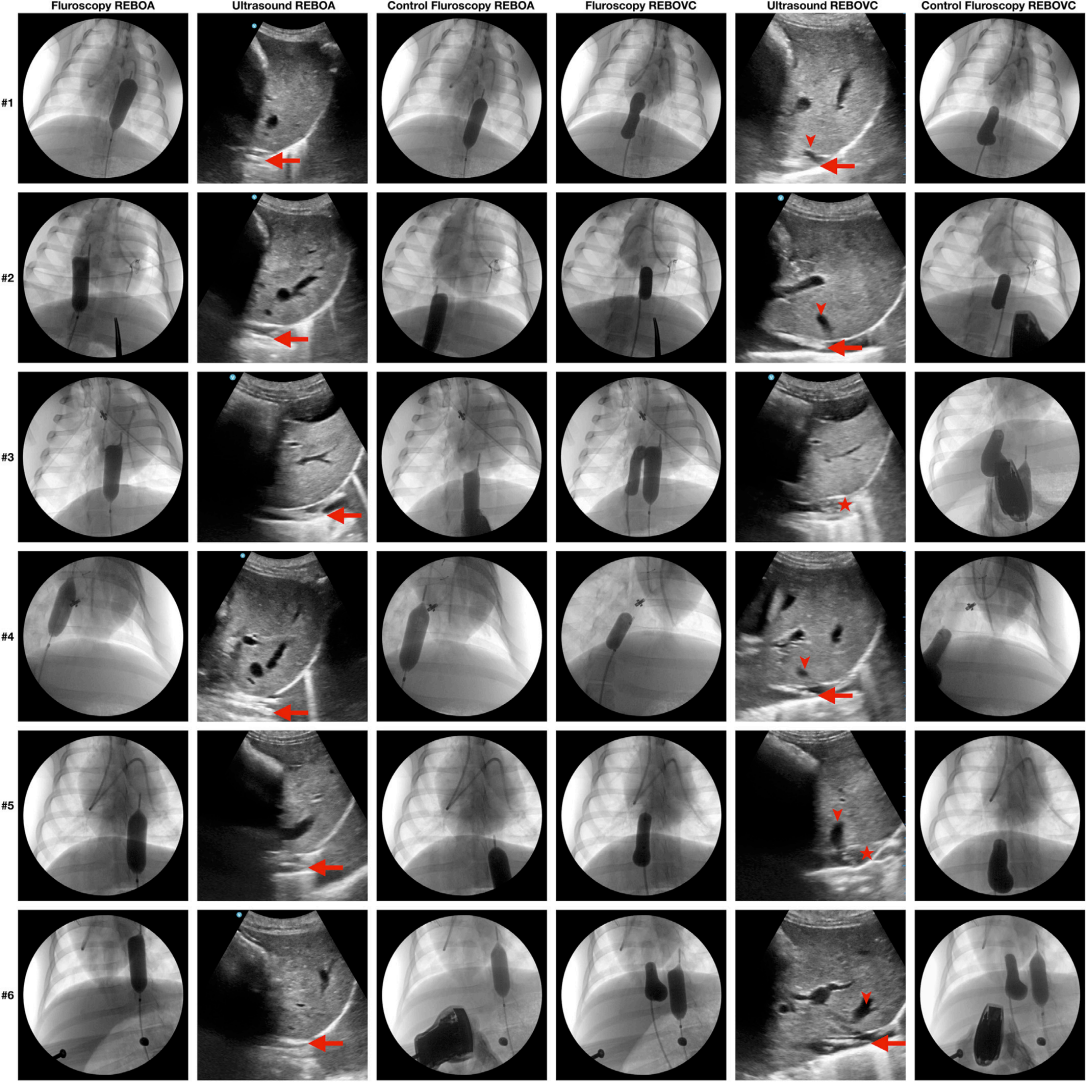
Fluoroscopic and ultrasonic images of resuscitative endovascular balloon occlusion of the supraceliac aorta (REBOA) or of the suprahepatic vena cava (REBOVC) placed either by fluoroscopy guidance or ultrasound guidance in six anesthetized pigs. Three of the animals (row #1–3) had REBOA and REBOVC fluoroscopy-guided placement first (columns 1 and 4) and thereafter ultrasound-guided placement (columns 2 and 5) which was confirmed by fluoroscopy (control fluoroscopy, columns 3 and 6). Three of the animals (row #4–6) had REBOA and REBOVC positioned by ultrasound guidance first which was confirmed by fluoroscopy, and second placement guided by fluoroscopy. Large arrows show the tip of the REBOA/REBOVC catheter. Small arrows show one of the hepatic veins at their confluence with the inferior vena cava. Asterisks show the REBOVC balloon deployed in the inferior vena cava cranially to the confluence of the hepatic veins in the inferior vena cava.

After randomization to either fluoroscopy or ultrasound guidance first, the REBOA was placed first and the REBOVC second in all groups since our previous porcine research has demonstrated poor hemodynamic tolerance to REBOVC without pre-existing REBOA.^13 14^ No premeasurements by using anatomical external landmarks were performed. The intravascular end-tip of the femoral sheath was the predefined starting position of either the REBOA or the REBOVC balloon catheter. The predefined fluoroscopic end position in the aorta was at the level of the diaphragm with 2/3 of the balloon above the diaphragm and 1/3 below. The predefined fluoroscopic end position of the suprahepatic inferior vena cava balloon was at the level of the most cranially located hepatic vein, caudal to the right atrium and, when visible, the eustachian valve, ‘the valve of the inferior vena cava’.^26^ Three persons performed all the procedures and measurements. One person measured the time, the second performed the ultrasound exam and the third positioned the endovascular balloons. Time started when the tip of the endovascular catheter left the distal opening of the intravascular sheath in the common femoral artery or vein, and the position was guided by either ultrasound or fluoroscopy by looking at common screens. The endovascular balloons were positioned by a consultant surgeon. An anesthetist, with proficient experience with abdominal ultrasound usage, performed all the ultrasound exams. A vascular surgery resident measured the times. Time was recorded in seconds and stopped when the predefined end position in either aorta or inferior vena cava was reached. After ultrasound-guided placement of the REBOA or the REBOVC, verification of the endovascular balloon position was performed by fluoroscopy before deflation (figure 2). Fluoroscopy and ultrasound were compared. The fluoroscopic position was considered the reference. The endovascular balloons used were Equalizer (Boston Scientific, Ireland), used without guidewire guidance. To mimic the standard Focused Assessment Sonography for Trauma (FAST) examination, anterior abdominal subxiphoidal probe locations were used, rather than the transhepatic flank or back position. The subxiphoidal, initially transverse, view was used to image the inferior vena cava and the aorta by ultrasound with an abdominal probe. The transverse orientation was then shifted to a longitudinal, vertical view. The pigs were kept in a left lateral position. To optimize the imaging, adaptations of the depth and gain were continuously performed.

### Statistical analysis

No a priori power calculation was performed due to the lack of a pilot or published effect data. The Shapiro-Wilk test was performed to examine the data for normal distribution. Since part of the data was not normally distributed, and the number of observations were few, non-parametric statistics were used. The Kruskal-Wallis test was performed to investigate differences between groups in positioning time. If statistically significant, the Mann-Whitney U test was then performed post hoc for multiple comparisons between the groups and adjusted by Bonferroni correction for multiple testing.

## Results

Ten animals were initially used for the study. One was used for method development and not included in the final analysis. Of the remaining nine animals, five were randomized to ultrasound-guided placement first, four to fluoroscopy-guided placement first (figure 1).

*From the ultrasound-first group*, two animals had circulatory collapse after the balloon inflation in suprahepatic vena cava, which caused reduced preload and secondary cardiac arrest. One of those two animals survived the ultrasound-guided REBOA and REBOVC placement but became hemodynamically unstable thereafter and did not survive until the following fluoroscopy examination. The other one survived ultrasound-guided placement of REBOA and REBOVC, then the fluoroscopy-guided REBOA placement. Thereafter, the animal died and the last examination—fluoroscopy-guided REBOVC placement—was therefore not performed in this animal.

*From the fluoroscopy-first group*, one animal had perforation of the vena cava during catheter insertion and died of hemorrhagic shock before the ultrasound-guided placements had commenced, that is, the ultrasound-guided REBOA and REBOVC were not performed in this animal. In total, eight animals finished fluoroscopy REBOA, ultrasound REBOA and ultrasound REBOVC, respectively, and seven animals completed fluoroscopy REBOVC measurements. All ultrasound-guided REBOAs (n=8) and REBOVCs (n=8) were accurately positioned by ultrasound, as verified by fluoroscopy (figure 2).

Attached to the manuscript are three video clips, the first showing ultrasound-guided supraceliac REBOA placement. The second video shows ultrasound-guided suprahepatic REBOVC placement. The third video illustrates inflation of the REBOVC balloon at the level of the hepatic veins in the inferior vena cava (online supplemental videos 1–3).

10.1136/tsaco-2022-001075.supp1 Supplementary video

10.1136/tsaco-2022-001075.supp2 Supplementary video

10.1136/tsaco-2022-001075.supp3 Supplementary video

**Figure 3:**
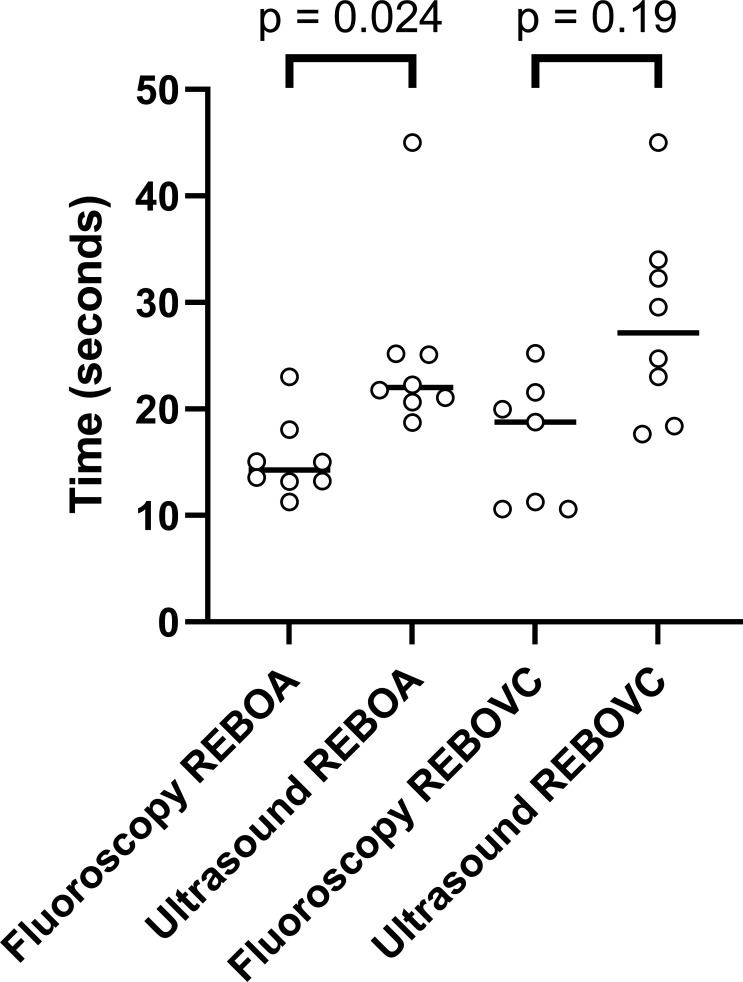
Time to positioning of resuscitative endovascular balloon occlusion of the supraceliac aorta (REBOA) or of the suprahepatic vena cava (REBOVC) either by fluoroscopy guidance or ultrasound guidance in nine anesthetized pigs (n=7–8 per intervention).

The median time to place REBOA was 14 s with fluoroscopy guidance and 22 s with ultrasound guidance (p=0.024). The median time to place REBOVC with fluoroscopy guidance was 19 s and with ultrasound guidance 28 s (p=0.19, figure 3).

## Discussion

This study suggests that suprahepatic REBOVC may quickly be guided to the correct position using ultrasound in a laboratory porcine model. To our knowledge, no scientific article has previously described the use of ultrasound for the guided positioning of REBOVC. Case reports where REBOVC has been used as a bridge to surgery in hemorrhagic shock and patients in extremis have been published.^3 4 27^ A recent report from Baltimore, presenting the use of REBOVC in five trauma patients, provides support for successful clinical use of REBOVC in the management of vena cava injuries.^5^ The use of REBOVC as an adjunct to REBOA in vena cava injuries is a new tool, recommended under very special circumstances. REBOVC may be considered a tool for the hybrid management of vena cava injuries, where vascular control is achieved by combining endovascular techniques with definitive open surgical repair.^12^ Similar to the REBOA, for the REBOVC to be a clinically available tool, it must be able to be safely, quickly and correctly positioned. In trauma patients, ultrasound provides a rapid, cheap and non-invasive tool.

The subxiphoidal view is one of the standard FAST examination views and although examination of the vena cava is not part of the standard FAST protocol, the inferior vena cava can relatively easily be seen by anyone performing FAST exams with some training.^28^ In a pilot study by Martin *et al*, identifying the inferior vena cava by ultrasound was shown to be a skill that is relatively fast to learn.^28^ Thus, we believe that positioning REBOVC guided by ultrasound may be a skill that can be quickly learned.

For placement of REBOA in less emergent situations, catheter insertion by guidewire and balloon inflation is often guided by fluoroscopy to optimize the position.^15^ However, in rural emergency departments, prehospital settings and austere military environments, fluoroscopy may not be available.^29^ In such settings, external anatomical landmarks or standard fixed distance models can guide REBOA placement.^15 18 30 31^ However, blind placement methods have their limitations.^31^ Differences in habitus, age, current physiology and pregnancy, vessel morphology and intravascular distances may alter the representativity and the reliability of anatomic landmarks and fixed distances.^15–18^ Even with the help of guidewires, blind REBOA advancement to the correct position in the vessel may be difficult.^17^ Incorrect balloon positioning may have devastating consequences.^21^ Therefore, if possible, radiological or sonographic confirmation of proper balloon placement is recommended for all levels of endovascular balloon placement.^17^ One animal died of vena cava perforation during catheter insertion. An intravascular resistance was felt at initial catheter insertion but was forced and unintentionally possibly caused the vessel injury. Vessel wall damage is a known complication to all arterial and venous endovascular management, and a risk associated with the method. Venous vessels are thinner, more fragile and may more easily be hurt, which must be considered when dealing with venous endovascular catheters.^8 32^ That incident happened despite guidance by fluoroscopy. However, considering the limited clinical experience with REBOVC and the results of this study, we do not recommend placement of REBOVC without fluoroscopy in humans. If available, combined usage of ultrasound and portable fluoroscopy would be advisable.

Since endovascular balloon occlusion of the vena cava is a new method to manage inferior vena cava injuries, no ‘standard balloons’ are available. Our choice of the Equalizer balloon was based on our previous experience with this balloon for vena cava occlusion. The Equalizer balloon is made of natural rubber latex, which makes it highly compliant and suitable for suprahepatic venous occlusion.^14^ This balloon requires a 7 Fr sheath, comparable to other commonly used endovascular balloons, such as the ER-REBOA (Prytime Medical, Texas, USA). However, we used a 10 Fr sheath and thus avoided balloon placement with guidewire insertion, not to facilitate visualization.

The median placement times for fluoroscopy-guided and ultrasound-guided REBOA and REBOVC was short. Importantly, this time does not include gaining femoral access, only balloon positioning. The time differences between fluoroscopy-guided REBOVC and ultrasound-guided REBOVC were small and may be considered negligible in a clinical setting. The number of animals included was small and the study was performed in a controlled laboratory environment, which must also be considered when evaluating the time measurements. In a chaotic emergency setting, the time taken to perform ultrasound-guided or fluoroscopy-guided endovascular placement may differ considerably. In our laboratory, the imaging equipment was set up and ready from start, while in the trauma bay, the ultrasound or fluoroscopy must be set up before examination. However, the primary goal of the study was not to perform a time comparison between ultrasound and fluoroscopy but rather to examine if ultrasound could be used to guide REBOVC placement. The anesthetist performing the ultrasound exam in this study was considered representative to the potential medical staff that may perform ultrasound exams in trauma patients in prehospital and intrahospital settings.

### Limitations

This study has some limitations. Although it is based on a porcine model and its applicability to human anatomy is not proven, the vascular anatomy of the pigs is similar to humans with regard to the inferior vena cava and the aorta.^33^ Pigs have five main hepatic veins, whereas humans have three. Similar to humans, these five porcine hepatic veins have two main drainage locations in the cranial third of the retrohepatic vena cava.^34^ Through our own experience, we have assessed the porcine suprahepatic vena cava to be similar in length to the corresponding human vessel. Unlike humans, the entry of inferior vena cava to the right atrium is not easily visualized by ultrasound in pigs. However, a too cranially located REBOVC should be seen by ultrasound in humans. No other known major anatomical differences are regarded to have impacted the results in this study.^35^ In humans, obesity and gas-filled bowels may make visualization by ultrasound more challenging or even impossible.^21 36 37^ The pigs had a mean weight of 30 kg and thus may be comparable to human children in terms of weight and abdominal wall configuration. The laboratory has a long-time cooperation with this farmer, providing this special breed of pigs. Even though we could have used heavier pigs, at the time of laboratory research, these animals were the ones provided by the local farmer. Our study was performed in normovolemic conditions and not in a state of hemorrhagic shock physiology, when REBOVC is most likely to be used. In hemorrhagic shock, the blood vessels may be more difficult to visualize due to lumen collapse, vasospam and other effects of the cathecholamine response.^17^ With any endovascular manipulation, there is a risk of injury to the vessel wall. Since venous vessels generally are thinner and more fragile, insertion and inflation of endovascular balloons in veins warrant extra caution. Trauma patients may have occult vessel injuries. Insertion of endovascular catheters in such vessels can worsen those injuries and may be fatal.^17 38^

## Conclusion

This study suggests that subxiphoidal ultrasound can be used to quickly guide the placement of the REBOVC catheter into the suprahepatic inferior vena cava position in a porcine laboratory model; however, the combined use with fluoroscopy probably increase safety. Studies in a controlled human setting are needed before application of this method in trauma patients.

## Data Availability

Data are available on reasonable request.
